# A Machine Learning Model to Aid Detection of Familial Hypercholesterolemia

**DOI:** 10.1016/j.jacadv.2023.100333

**Published:** 2023-05-24

**Authors:** Jasmine Gratton, Marta Futema, Steve E. Humphries, Aroon D. Hingorani, Chris Finan, Amand F. Schmidt

**Affiliations:** a Institute of Cardiovascular Science, University College London, London, United Kingdom; b Cardiology Research Centre, Molecular and Clinical Sciences Research Institute, St George’s University of London, London, United Kingdom; c UCL British Heart Foundation Research Accelerator; d Health Data Research UK, London, United Kingdom; e Division Heart and Lungs, Department of Cardiology, University Medical Centre Utrecht, Utrecht University, Utrecht, the Netherlands

**Keywords:** FH, polygenic score, prediction, screening, UK Biobank

## Abstract

**Background:**

People with monogenic familial hypercholesterolemia (FH) are at an increased risk of premature coronary heart disease and death. With a prevalence of 1:250, FH is relatively common; but currently there is no population screening strategy in place and most carriers are identified late in life, delaying timely and cost-effective interventions.

**Objectives:**

The purpose of this study was to derive an algorithm to identify people with suspected monogenic FH for subsequent confirmatory genomic testing and cascade screening.

**Methods:**

A least absolute shrinkage and selection operator logistic regression model was used to identify predictors that accurately identified people with FH in 139,779 unrelated participants of the UK Biobank. Candidate predictors included information on medical and family history, anthropometric measures, blood biomarkers, and a low-density lipoprotein cholesterol (LDL-C) polygenic score (PGS). Model derivation and evaluation were performed in independent training and testing data.

**Results:**

A total of 488 FH variant carriers were identified using whole-exome sequencing of the *low-density lipoprotein receptor*, *apolipoprotein B, apolipoprotein E, proprotein convertase subtilisin/kexin type 9* genes. A 14-variable algorithm for FH was derived, with an area under the curve of 0.77 (95% CI: 0.71-0.83), where the top 5 most important variables included triglyceride, LDL-C, apolipoprotein A1 concentrations, self-reported statin use, and LDL-C PGS. Excluding the PGS as a candidate feature resulted in a 9-variable model with a comparable area under the curve: 0.76 (95% CI: 0.71-0.82). Both multivariable models (w/wo the PGS) outperformed screening-prioritization based on LDL-C adjusted for statin use.

**Conclusions:**

Detecting individuals with FH can be improved by considering additional predictors. This would reduce the sequencing burden in a 2-stage population screening strategy for FH.

Familial hypercholesterolemia (FH) is an autosomal dominant disorder caused by variants in the low-density lipoprotein receptor (*LDLR*), apolipoprotein B (*APOB*), proprotein convertase subtilisin/kexin 9 (*PCSK9*), or apolipoprotein E (*APOE*) genes. It is characterized by elevated low-density lipoprotein cholesterol (LDL-C) concentration and premature coronary heart disease (CHD).^1^ FH-causing variants are found in about 1 in 250 individuals (95% CI: 1:345-1:192)^2^; however, the condition remains highly underdiagnosed worldwide with only an estimated 1% to 10% of cases diagnosed.^3^^,^^4^ Affected individuals are at increased risk of premature CHD, due to lifelong exposure to elevated levels of LDL-C, where early initiation of lipid-lowering treatment is paramount for risk management.^3^ There is currently no systematic way of identifying new index FH cases in the general adult population in most countries (eg, in the United Kingdom (UK) and United States), although, cascade testing in families of affected individuals has been shown to be highly cost-effective.^5^, ^6^, ^7^, ^8^ Currently, patient diagnosis often happens after the development of CHD symptoms or by opportunistic measurement of lipid profile and at the discretion of clinicians. Diagnosis is made using tools such as the Dutch Lipid Clinical Network and the Simon Broome criteria, which have not been designed to be used as population screening tools.^1^

In 2016, Wald et al^9^ suggested screening children aged 15 months of age by measurement of total or LDL-C to systematically identify index monogenic FH cases in the general population as a prelude to testing parents and other family members. Futema et al^10^ showed that measurement of LDL-C alone at age 9 may be insufficiently accurate in reliably distinguishing FH-variant carriers from those with an elevated cholesterol as a consequence of diet and lifestyle factors or carriage of a high burden of common cholesterol-raising alleles, and suggested adding a confirmatory targeted-sequencing step to reduce the number of false positive cases detected.

The increased availability of routine health checks in adults either through work-place schemes or local healthcare providers offers an opportunity to systematically identify adult carriers of FH-causing variants.^11^ Positioning adult FH screening within routine health checks, which typically record a substantial number of other clinical measurements, offers the opportunity to consider additional predictors for FH. This may be important, because, while the effect of FH on CHD risk is mediated through elevated circulating LDL-C concentration, it is well-known that LDL-C concentration associates with other variables such as blood and liver biomarkers, diet, and also with common genetic variants.^12^ Combining multiple environmental factors and a polygenic score (PGS) for LDL-C raising genetic variants may improve the detection of people with monogenic FH for prioritization for confirmatory genetic testing.^13^^,^^14^ This is because individuals with monogenic FH are likely to have a measured LDL-C concentration that is higher than can be accounted for by these other variables.

In the current manuscript we utilize the UK Biobank data to evaluate the detection rate and testing burden of 4 prioritization strategies to identify people with suspected FH-causing variants for confirmatory genetic testing: 1) no prioritization (ie, referring all participants for sequencing); 2) a plasma LDL-C-based prioritization model adjusting for statin treatment; 3) a multivariable machine learning prioritization model with nongenetic variables; and 4) a multivariable machine learning prioritization model which includes a PGS for LDL-C (Central Illustration).

**Central Illustration:**
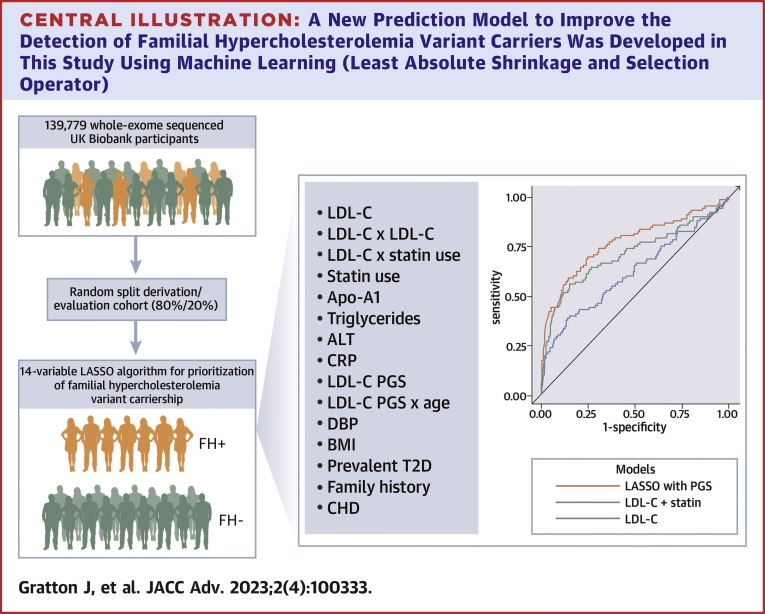
**A New Prediction Model to Improve the Detection of Familial Hypercholesterolemia Variant Carriers Was Developed in This Study Using Machine Learning (Least Absolute Shrinkage and Selection Operator)** This model improves the prioritization of individuals for familial hypercholesterolemia–variant genomic sequencing confirmation. The model, developed and derived in the UK Biobank (with 139,779 whole-exome sequenced participants including 488 familial hypercholesterolemia variant carriers), included 14 predictor variables such as low-density lipoprotein cholesterol, apolipoprotein A1, triglyceride, alanine aminotransferase, c-reactive protein concentrations, statin use, low-density lipoprotein cholesterol polygenic score, age, diastolic blood pressure, body mass index, prevalent type 2 diabetes, family history of coronary heart disease, and interaction terms. It performed better than a model using low-density lipoprotein cholesterol or low-density lipoprotein cholesterol and statin use only. The **green** icons represent unaffected individuals, while those in **orange** represent familial hypercholesterolemia carriers. ALT = alanine aminotransferase; Apo-A1 = apolipoprotein A1; BMI = body mass index; CHD = coronary heart disease; CRP = C-reactive protein; DBP = diastolic blood pressure; LASSO = least absolute shrinkage and selection operator; LDL-C = low-density lipoprotein cholesterol; PGS = polygenic score; T2D = type 2 diabetes.

## Methods

### Available genomics data and FH ascertainment

We identified 472,147 UK Biobank participants of White British ancestry (data-field 21000) as part of the approved project identifications 40721 and 44972. After performing genomic quality control steps (Supplemental Appendix), 341,515 individuals remained, including 140,439 with whole-exome sequencing (WES) data necessary to identify those who carry an FH-causing variant. Causal FH variants were searched for in the WES data encompassing the *LDLR*, *APOB*, and *PCSK9* genes (Supplemental Appendix, Supplemental Table 1). The pathogenic variant p.Leu167del in *APOE* associated with FH was extracted.^15^ A total of 488 pathogenic and likely pathogenic FH variants were identified (Supplemental Table 2). Additionally, 660 participants were found to carry FH variants of uncertain significance (VUS) (Supplemental Table 3). These were excluded from the analysis because more evidence is required to interpret the effect of those VUS.

### LDL-C PGS generation

We next generated a PGS for LDL-C concentration using an independent data subset of 173,672 White British participants without lipid-lowering medication or WES data (Supplemental Figure 1). An initial list of 10,137 genetic variants with a *P* value threshold of <5 × 10^−4^ was obtained from the Global Lipids Genetics Consortium genome-wide association study summary statistics for LDL-C.^16^ To reduce the number of potentially redundant variants and optimize LDL-C prediction, we next applied a least absolute shrinkage and selection operator (LASSO) regression algorithm using the biglasso package in R.^17^ The degree of penalization was determined through 15-fold cross-validation, maximizing the explained variance (R^2^), which resulted in a 1,466 genetic variant LDL-C PGS.

### Deriving machine learning algorithms to prioritize participants with FH

We extracted data on a total of 24 candidate predictors related to FH, and feasible to measure and obtain in a clinical setting, specifically: LDL-C, high-density lipoprotein cholesterol (HDL-C), total cholesterol, triglycerides, lipoprotein A, apolipoprotein A1 (Apo-A1), apolipoprotein B, C-reactive protein (CRP), aspartate aminotransferase, alanine aminotransferase (ALT), alkaline phosphatase, sex, body mass index (BMI), age, self-reported statin use, alcohol use, systolic blood pressure, diastolic blood pressure (DBP), Townsend deprivation index, smoking status, family history of CHD, type 2 diabetes diagnosis, hypertension, and LDL-C PGS. This was expanded by including 10 product terms between: age and LDL-C, age and LDL-C PGS, LDL-C PGS and LDL-C, age^2^, LDL-C^2^, statin use and LDL-C, family history of CHD and sex, family history of CHD and statin use, family history of CHD and alcohol use, and family history of CHD and hypertension. The limited missing data (Supplemental Table 4) were singly imputed using the R package MICE.^18^

Model derivation was performed using the WES data, applying a 80% training data split of 111,824 subjects, retaining 20% testing data (containing 93 carriers of 27,955 subjects) to unbiasedly evaluate model performance (Supplemental Figure 1, Supplemental Table 5). To prevent potential model instability, highly correlated variables (ie, multicollinear) were removed. These included apolipoprotein B and total cholesterol (Supplemental Figure 2). Variables were standardized to mean 0 and standard deviation (SD) 1 (Supplemental Tables 6 and 7). Finally, we applied a binomial regression model with LASSO penalization to derive a discrimination–optimized FH prediction model. Specifically, optimal penalization was determined through 15-fold cross-validation maximizing the area under the receiver operating characteristic curve (AUC).^17^ A first multivariable model was derived with nongenetic variables only (ie, without LDL-C PGS), and a second model was generated with the inclusion of LDL-C PGS.

Model performance was evaluated using the 20% testing data based on its discriminative ability (AUC), appropriate calibration of predicted and observed probability of having an FH variant (using calibration plots, calibration-in-the-large [CIL], and calibration slope [CS]), and classification metrics (sensitivity, specificity (or its compliment the false positive rate), positive predictive value, and the negative predicted value).

### Model comparison: a decision curve analysis

A decision curve analysis was performed using dcurves package version 0.3.0,^19^ comparing a model’s net benefit across various probability thresholds for confirmatory FH screening. Here, “net benefit” is calculated as the weighted difference between true and false positives at a specific threshold.^20^ A decision curve analysis is preferable to a net reclassification analysis as it provides a comparison of all models over all possible probability thresholds, instead of focusing on 1 arbitrary threshold.

### Evaluating the burden of genomic sequencing for FH

While genetic sequencing is the gold standard for FH diagnosis, it may often be prohibitively expensive to offer it to an entire population as a screening strategy. We, therefore, explored whether prioritizing people with suspected FH can reduce the screening burden with an acceptable number of false-negative results. We evaluated the following prioritization strategies: 1) no prioritization (ie, referring all participants for sequencing); 2) prioritization based on LDL-C concentration (adjusting for statin use); 3) a multivariable model built from clinical biomarkers and environmental predictors only, 4) a multivariable model built from genetic, clinical biomarkers, and environmental predictors. These prioritization strategies were evaluated on the number of subjects that would need to be sequenced, the proportion of FH carriers who would be missed, and the ratio of FH carriers correctly prioritized by the number of noncarriers unnecessarily offered sequencing. We further wish to emphasize that the 0.006 probability threshold was selected as an example to illustrate the potential impact of cascade screening. While this threshold was supported by our decision curve analysis (ie, located within the plausible range of probability thresholds), more formal analyses of cost and benefit need to be conducted before deciding on a more definitive threshold relevant for clinical implementation.

## Results

### Participant characteristics of our study cohort

Using the UK Biobank WES data, we identified 488 pathogenic or likely pathogenic FH variant carriers (list of variants shown in Supplemental Table 2) and 139,291 noncarriers; prevalence of 0.35% (95% CI: 0.32-0.38). FH variant carriers had a significantly higher frequency of a family history of CHD (62.7% vs 48.1% in controls), higher prevalence (8.2% vs 2.8% in controls) and incidence (6.6% vs 3.9% in controls) of CHD (Table 1).

**Table 1:** UK Biobank Participant Characteristics Stratified by Carrying a FH-Causing Variant

	FH-Variant Negative (n = 139,291)	FH-Variant Positive (n = 488)	*P* Value	Missing (%)
Male	63,382 (45.5)	207 (42.4)	0.187	0.0
Age (y)	58.0 (51.0–63.0)	58.0 (51.0–63.0)	0.803	0.0
Townsend deprivation index	−2.4 (−3.8 to 0.0)	−2.2 (−3.7 to 0.2)	0.346	0.1
BMI, kg/m^2^	26.7 (24.1–29.8)	27.1 (23.9–29.8)	0.689	0.3
Smoking status			0.685	3.7
Non-smoker	76,862 (57.3)	262 (56.2)		
Former smoker	49,302 (36.7)	171 (36.7)		
Light smoker (<10 cigarettes/d)	1,952 (1.5)	6 (1.3)		
Moderate smoker (10-19 cigarettes/d)	3,296 (2.5)	13 (2.8)		
Heavy smoker (>20 cigarettes/d)	2,796 (2.1)	14 (3.0)		
Alcohol use (%)			0.492	0.0
Prefer not to answer	88 (0.1)	1 (0.2)		
1/d	29,719 (21.3)	93 (19.1)		
3-4 times/wk	34,015 (24.4)	135 (27.7)		
1-2 times/wk	36,823 (26.4)	130 (26.6)		
1-3 times/mo	15,498 (11.1)	54 (11.1)		
Special occasions	14,383 (10.3)	45 (9.2)		
Never	8,765 (6.3)	30 (6.1)		
Family history of CHD	67,013 (48.1)	306 (62.7)	<0.001	0.0
Systolic blood pressure, mm Hg	136.5 (125.0–149.5)	135.0 (124.5–148.5)	0.119	0.2
Diastolic blood pressure, mm Hg	82.0 (75.0–89.0)	81.0 (74.0–87.0)	0.024	0.2
Statin use	18,139 (13.0)	165 (33.8)	<0.001	0.0
Hypertension	7,946 (5.7)	35 (7.2)	0.195	0.0
LDL-C PGS	3.7 (3.5–3.9)	3.7 (3.5–3.9)	0.652	0.0
Biomarkers				
LDL-C (unadjusted for statin use), mmol/L	3.5 (3.0–4.1)	3.9 (3.2–4.9)	<0.001	5.0
HDL-C, mmol/L	1.4 (1.2–1.7)	1.4 (1.2–1.6)	0.086	12.5
Total cholesterol, mmol/L	5.7 (4.9–6.4)	6.1 (5.2–7.3)	<0.001	4.8
Lipoprotein(a), nmol/L	20.0 (9.3–59.8)	27.6 (10.3–59.2)	0.083	24.3
Apolipoprotein A1, g/L	1.5 (1.4–1.7)	1.5 (1.3–1.6)	<0.001	13.0
Apolipoprotein B, g/L	1.0 (0.9–1.2)	1.2 (1.0–1.4)	<0.001	5.3
Triglycerides, mmol/L	1.5 (1.1–2.2)	1.3 (0.9–1.9)	<0.001	4.9
C-reactive protein, mg/L	1.3 (0.7–2.7)	1.2 (0.6–2.3)	0.065	5.1
Aspartate aminotransferase, U/L	24.4 (21.0–28.8)	25.1 (21.0–29.6)	0.111	5.2
Alanine aminotransferase, U/L	20.1 (15.4–27.3)	20.2 (15.6–27.2)	0.848	4.9
Alkaline phosphatase, U/L	80.1 (67.1–95.4)	80.6 (66.8–96.1)	0.506	4.8
Disease prevalence and incidence				
CHD prevalence	3,890 (2.8)	40 (8.2)	<0.001	0.0
CHD incidence	5,370 (3.9)	32 (6.6)	0.003	0.0
CVD prevalence	5,686 (4.1)	45 (9.2)	<0.001	0.0
CVD incidence	9,038 (6.5)	46 (9.4)	0.011	0.0
Type 2 diabetes prevalence	3,593 (2.6)	11 (2.3)	0.757	0.0
Type 2 diabetes incidence	4,948 (3.6)	19 (3.9)	0.776	0.0

Values are n (%) or median (IQR). The *P* values shown in the table are from the Kruskal-Wallis Rank Sum test for continuous variables and from the Mann-Whitney *U* test for binary variables.

BMI = body mass index; CHD = coronary heart disease; CVD = cardiovascular disease; FH = familial hypercholesterolemia; HDL-C = high-density lipoprotein cholesterol; LDL-C = low-density lipoprotein cholesterol; PGS = polygenic score.

### Multivariable machine learning models to prioritize FH variant carriers

Nine nongenetic variables were retained by the LASSO regression model which did not include the LDL-C PGS (Supplemental Table 6). These predictors were age, statin use, systolic blood pressure, DBP, Apo-A1 and triglyceride concentrations, family history of CHD, and 2 interaction terms: LDL-C^2^, and statin use and LDL-C. Retention of these product terms indicated the presence of nonlinear associations with FH, eg, the LDL-C association with the presence of a monogenic FH variant was found to be quadratic (Supplemental Figure 4). The test data AUC for this model was of 0.76 (95% CI: 0.71-0.82).

**Figure 1:**
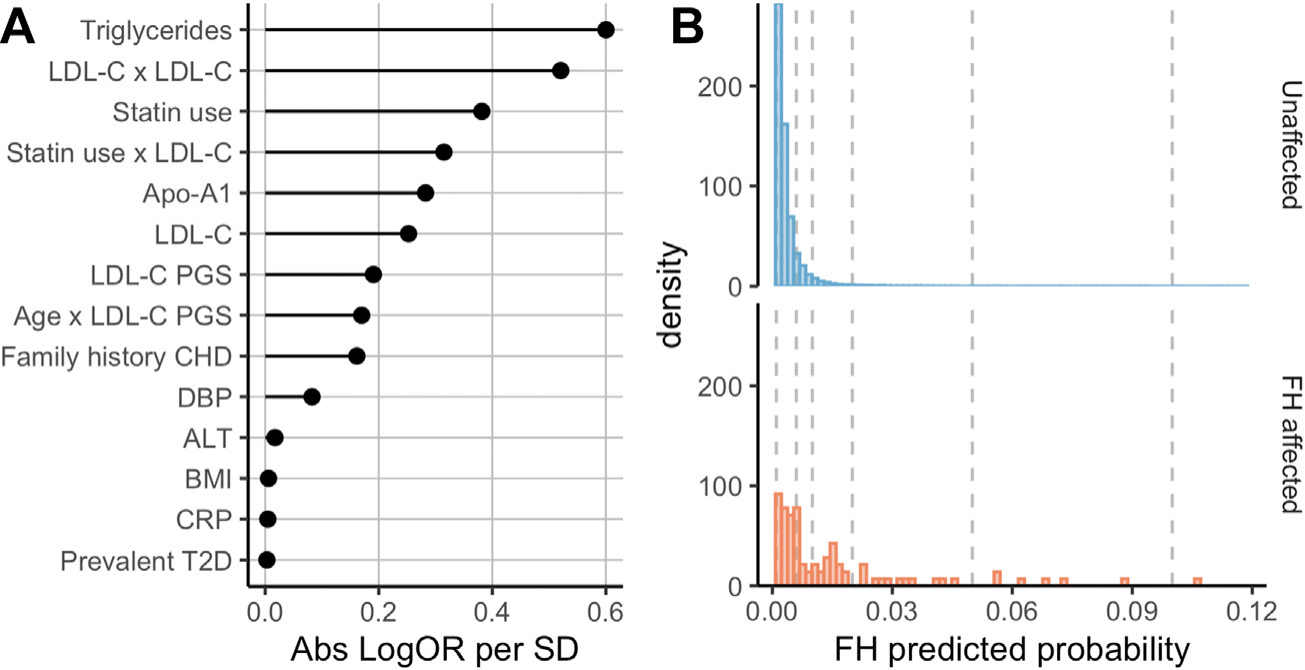
**Feature Importance of the Variables Retained by LASSO Regression Predicting Monogenic FH, and the Density Predicted Probability Distributions From This Model for Unaffected and Affected FH Individuals in White British Participants of the UK Biobank** **(A)** The 14 predictors retained by least absolute shrinkage and selection operator regression ordered by absolute log odds ratio per standard deviation. The **“x” sign** is used to indicate an interaction term. **(B)** The density predicted probability distributions for affected **(orange)** and unaffected **(blue)** FH participants in our test cohort as predicted by the multivariable model. Fourteen unaffected individuals had a monogenic FH predicted probability above 0.12 and are not shown on the plot for legibility purposes. The **vertical dotted lines** represent the various FH predicted probability thresholds evaluated in Supplemental Table 8. Abs = absolute; ALT = alanine aminotransferase; Apo-A1 = apolipoprotein A1; BMI = body mass index; CHD = coronary heart disease; CRP = C-reactive protein; DBP = diastolic blood pressure; FH = familial hypercholesterolemia; LDL-C = low-density lipoprotein cholesterol; PGS = polygenic score; T2D = type 2 diabetes.

**Figure 2:**
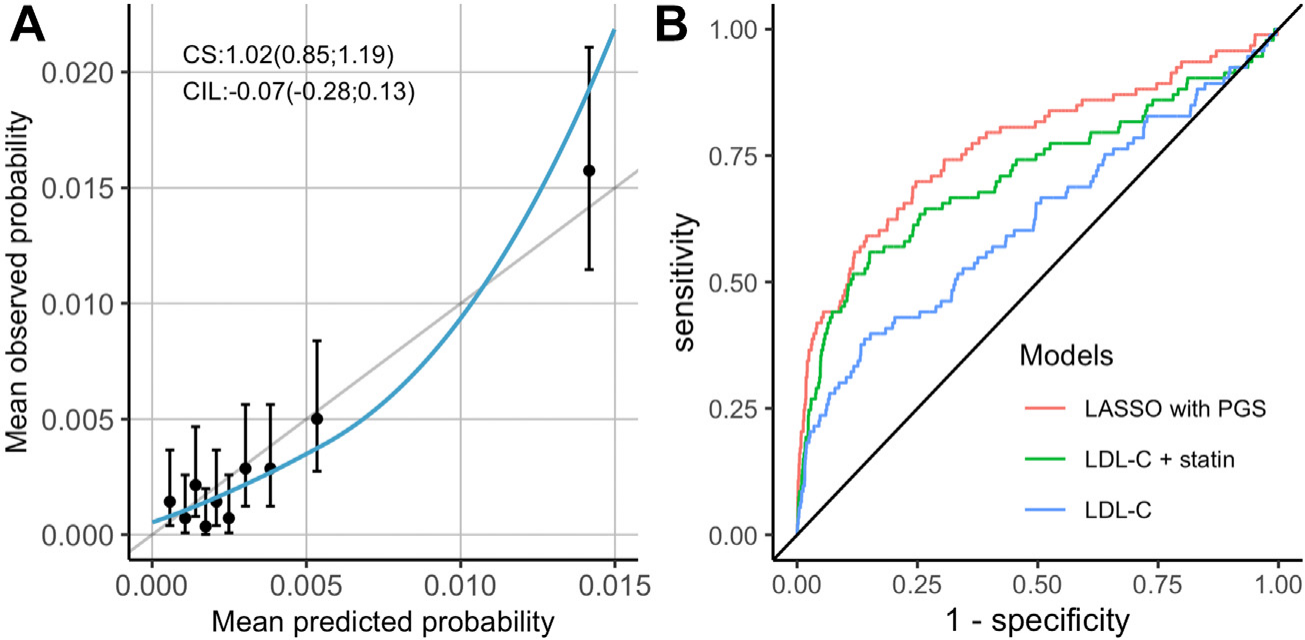
**Discrimination and Calibration of a Multivariable Algorithm Predicting FH Carriership Using Independent Testing Data** **(A)** The calibration plot for the multivariable model where the mean predicted and mean observed probability for each decile of the test data are depicted by the datapoints with their 95% CI. Perfect calibration is indicated by the **vertical gray line**. The calibration-in-the-large and the calibration slope values are indicated on the plot with their 95% CI in brackets. The loess line was fitted with familial hypercholesterolemia–causing variant status as the outcome and mean predicted probability as the predictor. **(B)** The receiver operating characteristic curves for the multivariable model **(red)**, LDL-C and statin model **(green)**, and LDL-C concentration only model **(blue)**. The area under the curve for each of these models are 0.77 (95% CI: 0.71-0.83), 0.71 (95% CI: 0.65-0.77), and 0.62 (95% CI: 0.56-0.68) respectively. CI = confidence interval; CIL = calibration-in-the-large; CS = calibration slope; LASSO = least absolute shrinkage and selection operator; LDL-C = low-density lipoprotein cholesterol; PGS = polygenic score.

Fourteen of the 32 variables were retained by the LASSO regression model which included a LDL-C PGS for the prediction of FH (Figure 1A, Supplemental Figure 3, Supplemental Table 7), including triglyceride, Apo-A1, ALT, and CRP concentrations, statin use, LDL-C PGS, family history of CHD, DBP, BMI, and prevalent type 2 diabetes. Additionally, the following product terms were selected: LDL-C^2^, statin use and LDL-C, and age and LDL-C PGS. The test data AUC for this model were comparable but superior to the previous model: 0.77 (95% CI: 0.71-0.83), with a training data AUC of 0.78 (95% CI: 0.75-0.81). Calibration statistics (calibration-in-the-large: −0.073 [95% CI: −0.28 to 0.13] and calibration slope: 1.02 [95% CI: 0.85-1.19]) indicated the predicted probability agreed well with the observed probability (Figure 2A). The median predicted probability of having monogenic FH by this multivariable model was ∼3-fold higher in FH carriers (0.64% [IQR: 0.31%-1.62%]) compared to noncarriers (0.23% [IQR: 0.14%-0.38%]), with partial overlap between FH carriers and noncarriers (Figure 1B).

Both multivariable machine learning models outperformed a model which only considered LDL-C (AUC: 0.62 [95% CI: 0.56-0.68]), as well as a model which corrected for statins (AUC: 0.71 [95% CI: 0.65-0.77]) (Figure 2B).

### Sensitivity analyses

We further investigated whether the model was better at predicting *APOB* or *LDLR* FH-causing variants. Using the test data, the AUC for predicting *APOB* FH-causing variants (which in 98% of the cases was the p.Arg3527Gln change) was 0.81 (95% CI: 0.69-0.94), and for predicting *LDLR* FH-causing variants was 0.76 (95% CI: 0.70-0.82). Additionally, we explored model performance across age groups (Supplemental Results), which did not differ significantly.

Finally, we additionally considered a model with HDL-C concentration instead of Apo-A1, where the former is more readily available in most clinical settings; also finding comparable performance with our original multivariable model (test data AUC of 0.77 [95% CI: 0.71-0.82]).

### Evaluating the FH sequencing strategies through decision curve analysis

**Figure 3:**
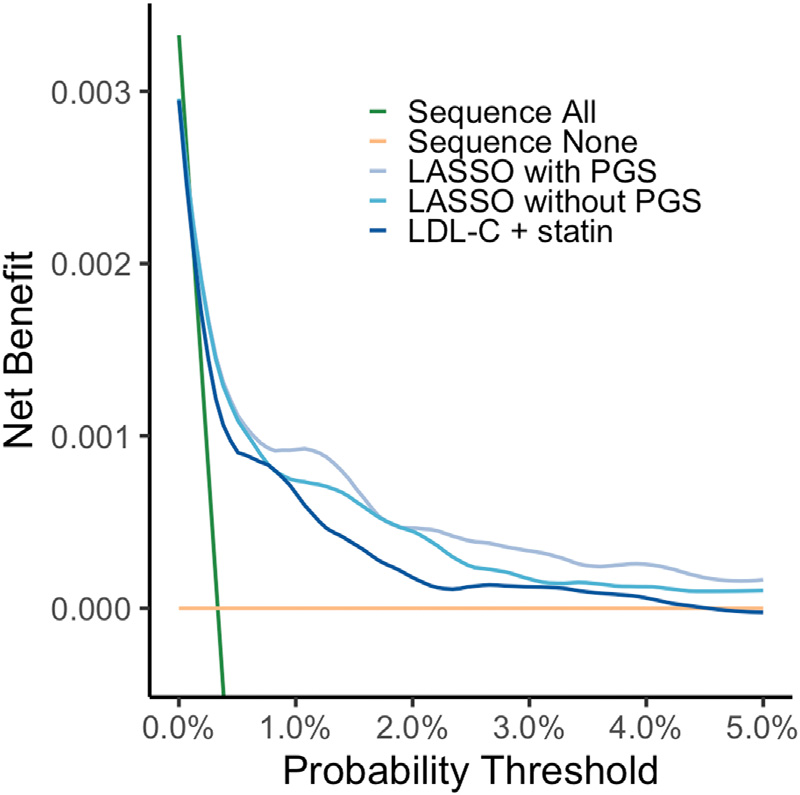
**Decision Curve Analysis of the Multivariable Models** The highest curve indicates the highest net benefit which considers the benefits and harms of a model. Sequence all refers to screening and sequencing the entire population, while sequence none refers to no familial hypercholesterolemia screening. The LDL-C + statin model is a model based on LDL-C concentration adjusted for statin use. The LASSO models are the multivariable machine learning models that either included or excluded a LDL-C polygenic score. LASSO = least absolute shrinkage and selection operator; LDL-C = low-density lipoprotein cholesterol; PGS = polygenic score.

We next determined at which probability threshold the net benefit of the various models was larger than the “sequence all” strategy (Figure 3). The net benefit of the “sequence all” strategy was lower than that of the other models tested at a threshold of 0.0013 (0.13%). This implies that model-based prioritization for confirmatory FH sequencing is more beneficial if one decided to screen 1/0.0013 = 769 or more people to detected one FH case. Irrespective of the probability threshold, the multivariable machine learning models had a larger net benefit than the LDL-C adjusted for statin use model. At a threshold of 0.0050 (0.5%), the multivariable model with the LDL-C PGS had the largest net benefit out of all the models tested (Figure 3).

### Prioritizing individuals for FH genomic testing in a 2-stage population screening strategy

As an illustrative example, we evaluated the impact of a 2-stage population screen for FH where the second stage consisted of targeted sequencing of FH variants, comparing the multivariable model with a PGS to the statin-adjusted LDL-C model (Supplemental Figure 5, Supplemental Table 8). In this example, we employed a common probability threshold of 0.006 (0.6%), which falls within the plausible range found using the decision curve analysis (Figure 3). On average, 7 additional FH carriers would be detected for 100,000 individuals screened when using the multivariable model with LDL-C PGS compared to the LDL-C and statin use model. This multivariable model would refer 12,033 individuals (12%) for genetic sequencing, compared to 14,730 (15%) with the LDL-C and statin use model, resulting in an 18% reduction in genetic testing for this specific threshold.

Furthermore, if we assume that FH has a population prevalence of 1 in 286 (equal to our cohort’s prevalence) and that one FH case has on average 1.5 first-degree relatives ([2 children + 1 sibling]/2) who are also affected by FH (discovered through cascade testing),^21^ then overall one FH case would be identified for every ∼219 people screened when using the multivariable model with LDL-C PGS, compared to one FH case for every ∼228 individuals screened with the LDL-C and statin use model.

## Discussion

In the current manuscript, we derived a multivariable machine learning model to identify people with suspected FH for confirmatory DNA sequencing in the context of population screening. Using LASSO regression, we derived a 14-feature model consisting of LDL-C, Apo-A1, triglyceride, ALT and CRP concentrations, self-reported statin use, family history of CHD, DBP, BMI, type 2 diabetes diagnosis, 3-product terms, and an LDL-C PGS. The multivariable algorithm was able to discriminate between FH and non-FH carriers with an AUC of 0.77 (95% CI: 0.71-0.83), with good calibration; outperforming a simpler model consisting of LDL-C and an indicator for statin prescription, and a multivariable model without LDL-C PGS as a predictor. The model presented here could be considered as part of a national screening programme for FH (eg, integrated with national or local vascular health checks) in countries (or even industries) where motivation, resources, and infrastructure allow it.

Above a classification threshold of 0.0013 (0.13%), the multivariable algorithm that contained the LDL-C PGS showed the highest net benefit out of all the models tested (Figure 3), and was able to decrease the number of subjects referred to genetic sequencing (as an example: from 100,000 individuals without any prioritization, to 14,730 with prioritization using the LDL-C and statin use model, and to 12,033 with prioritization using the multivariable model for a predicted probability threshold of carrying a variant for monogenic FH of 0.006; equivalent to approximately a 18% decrease in individuals needed to be sequenced between the last 2 models [Supplemental Figure 5]). These differences become more significant if extrapolating the values to a population-wide scale comprising of millions of participants screened. The choice of screening method for FH is very much dependent on the threshold chosen (Figure 3) and on the resources available. This manuscript explored the differences in performance of possible FH screening strategies in adults, and our results provide support for opportunistic screening and seeding of cascade testing for FH using the multivariable algorithms derived here, which could be integrated within existing health checks offered to employers or local health care providers (eg, the National Health Service vascular checks in the UK).^11^

Previously, Banda et al^22^ used a machine learning method to detect monogenic FH cases from electronic health records. While their model showed an impressive AUC of 0.94, one of their most important features was referral to a cardiology clinic, which is in very close proximity to confirmatory FH testing, limiting the model’s utility as a prospective tool for FH diagnosis. Besseling et al^23^ developed a multivariable model to identify FH carriers validated in study participants consisting of FH cases and their relatives, again limiting applicability to the general population. Our model instead considers FH prioritization in a non–general practice-referred population and is more generalizable as a systematic population screening tool.

Our multivariable model included 3 terms for LDL-C (LDL-C itself, LDL-C squared, and an interaction with statin prescription), which combined makes it the most important predictor. Additionally, our model also identified novel predictors for FH such as triglyceride and Apo-A1 concentrations, with triglycerides having the largest absolute odds ratio per SD (0.60). In our study we find that FH carriers had significantly lower triglyceride concentrations than noncarriers (Table 1), which resulted in a negative association, indicating that triglyceride concentrations can be useful in discriminating between individuals who have hypercholesterolemia due to lifestyle factors or other causes (eg, combined hyperlipidemia) instead of an FH-causing variant. We also found that higher Apo-A1 concentrations, a protein found on HDL particles, was associated with a decreased probability of FH. Apo-A1 concentration can also be readily replaced by HDL-C concentration without impacting model performance as shown in the Supplemental Results. Finally, we note that our multivariable FH model retained a squared term for LDL-C, suggesting that LDL-C is not linearly related with carrying an FH variant, but rather has a quadratic relationship (Supplemental Figure 4).

The variables included in our multivariable algorithm should not be interpreted as causal risk factors for monogenic FH; they simply help to distinguish nonmonogenetic sources of variation in LDL-C concentrations from monogenic causes (as was discussed in more detail previously with triglyceride concentrations). This also provides the rational for including an LDL-C PGS in the model: a large discrepancy between predicted LDL-C concentrations (by the LDL-C PGS) and observed LDL-C concentrations might be indicative of FH carriership,^13^^,^^14^ demonstrated here by a negative coefficient for LDL-C PGS in the model (Supplemental Table 7). We note that a previous LDL-C PGS by Wu et al^24^ had a substantially larger R-squared (0.21 [95% CI: 0.20-0.22]) than reported here (0.14 [95% CI: 0.13-0.15]). Unlike Wu et al who identified genetic variants from an internal UK Biobank LDL-C genome-wide association study overlapping with the PGS training data, we identified variants based on an independent dataset from Global Lipids Genetics Consortium,^16^ guarding against overfitting through ‘data-leakage’ between the training and testing data sets and providing a more robust estimate of explained variance. Currently, PGS information is not routinely used or collected in clinical practice, which is why we also derived a multivariable model without LDL-C PGS, which did not meaningfully differ (Supplemental Table 6). Previous studies have suggested that PGS could be used to identify individuals with a rare variant for certain diseases, such as FH.^13^^,^^14^ Our study confirms the utility of the PGS for FH prioritization; however, given its correlation with environmental variables (eg, lipid levels), this genetic information can be readily replaced with information from nongenetic data.

### Study limitations

A study limitation to consider is the exclusion of individuals with VUS from our study cohort. There is conflicting evidence as to the causal effects of these VUS in FH. We anticipate that some are likely to be FH-causing while others are not, but more research is needed. As more VUS are classified as either FH-causing or not, the model can be readily updated to reflect our growing understanding of FH. Additionally, it is impossible to know whether some study participants have been genetically tested for carrying an FH variant, and whether they might have modified their behavior (eg, diet) following their diagnosis. This could potentially impact the accuracy of the multivariable model developed here; however, considering that only approximately 7% of FH cases have been diagnosed in the UK,^25^ this low number of diagnoses is unlikely to have a significant effect on the model and results presented here.

We have tested our multivariable model in a dataset which was independent from the training data, with no significant difference between training and testing AUC (difference of 0.01), suggesting limited model overfitting to the current sample. Nevertheless, we acknowledge that the chosen cohort might not be fully representative of the general population due to the older age of the participants, however, the prevalence of FH in this study (1:286) is similar to the estimated population prevalence of FH (1:250), and the models’ performance did not significantly differ between different age categories in our study.^2^ Considering the older median age of the UK Biobank participants and the health discrepancies observed between the UK Biobank and the general UK population,^26^ we suggest that this model is locally validated and updated before applying it to distinct settings. Model validation and recalibration should especially be conducted when considering populations of non-European ancestry. Irrespective of the important considerations regarding model transferability, prior to integrating the model in clinical care, an informed decision should be made on the optimal predicted probability threshold for monogenic FH classification (Figure 3). We wish to highlight that our choice of 0.006 as a threshold in Supplemental Figure 5 is simply an illustration, and depending on the available health care resources, a different threshold might be preferred (Figure 3).

A cost-benefit analysis would be informative in terms of whether the implementation of these models is cost-efficient. However, this type of analysis is country dependent and was not the aim of the current study. Nonetheless, we anticipate the additional cost to be marginal if taking into account the rapidly declining cost of genotyping, the fact that most of the biomarkers in the models are readily available in routine clinical practice, and that the infrastructure of screening facilities and programmes (eg, the National Health Service vascular checks in the UK) are already set up in many places.

## Conclusions

We derived a multivariable classification model for detecting monogenic FH variant carriers that outperformed a model based on LDL-C concentration (adjusted for statin use) for FH screening, and that offers an opportunity to prioritize suspected FH carriers for genetic sequencing.

### Code availability

The mean, SD, and coefficients of the variables are available in Supplemental Tables 6 and 7 for implementation.

PERSPECTIVES **COMPETENCY IN MEDICAL KNOWLEDGE:** FH carriers are at greater risk of premature CHD and death and are usually diagnosed late in life, missing the opportunity of disease prevention. The identification of index FH carriers can be improved and will enable further cascade testing in families.

**TRANSLATIONAL OUTLOOK 1:** The systematic identification of FH carriers earlier in life could improve the outcome of these patients.

**TRANSLATIONAL OUTLOOK 2:** The identification of FH carriers in a population has important implications in downstream cascade testing of close relatives who are at 50% risk of also carrying the pathogenic variant.

## Funding support and author disclosures

Dr Gratton is supported by the 10.13039/501100000274BHF studentship FS/17/70/33482. Drs Futema and Humphries were supported by a grant from the 10.13039/501100000274British Heart Foundation (BHF grant PG 08/008) and by funding from the Department of Health’s NIHR Biomedical Research Centers funding scheme. Dr Hingorani is a member of the advisory group for the Industrial Strategy Challenge Fund Accelerating Detection of Disease Challenge; a co-opted member of the National Institute for Health and Care Excellence Guideline update group for ‘cardiovascular disease: risk assessment and reduction, including lipid modification, CG181’; is a co-Investigator on a grant from 10.13039/100004319Pfizer to identify potential therapeutic targets for heart failure using human genomic and a collaborator on a grant from New Amsterdam Pharma; and is an NIHR Senior Investigator. Dr Finan has received funding from New Amsterdam Pharma for unrelated work; and received additional support from the National Institute for Health Research University College London Hospitals Biomedical Research Centre. Dr Schmidt is supported by the 10.13039/501100000274BHF grants PG/18/5033837 and PG/22/10989 and the UCL 10.13039/501100000274BHF Research Accelerator AA/18/6/34223; has received funding from 10.13039/501100011725Servier for unrelated work; has received funding from New Amsterdam Pharma for unrelated work; and has received additional support from the National Institute for Health Research University College London Hospitals Biomedical Research Centre. All other authors have reported that they have no relationships relevant to the contents of this paper to disclose.

## Footnotes

Appendix For supplemental methods, results, tables, and figures, please see the online version of this paper.
