# Supplementary material for: A Machine Learning Model to Aid Detection of Familial Hypercholesterolemia
Source: JACC Adv. 2023 May 24;2(4):100333. doi: 10.1016/j.jacadv.2023.100333 (PMC11198649; doi:10.1016/j.jacadv.2023.100333)

**SUPPLEMENTAL APPENDIX**

**SUPPLEMENTAL METHODS**

**Quality control of the genotyping data**

Genotyped individuals of European ancestry (data-field 22006) of the UK Biobank (application number 40721) underwent stringent quality control (QC) prior to analysis. Outliers for heterozygosity and missingness (data-field 22027) and putative chromosomal aneuploidy (data-field 22019) were excluded from the dataset. Individuals with sex mismatches between their self-reported sex and their genetic sex, and participants with missing genetic sex information were also excluded. Variant QC of the imputed genotype data was performed with bgenix,(1) and all genetic variants with an imputation information (INFO) score <0.3 were removed. For the QC of the remaining genetic variants, Bristol’s MRC-IEU protocol was followed: genetic variants were excluded if they had a minor allele frequency (MAF) <0.1%, MAF <0.5% and INFO <0.9, MAF <1% and INFO <0.8, MAF <3% and INFO <0.6.(2) Related individuals (up to and including third degree relatives) were also excluded from the analysis.

**FH case detection in the UK Biobank**

Multiallelic sites were converted to biallelic sites using BCFtools version 1.11.(3), and genetic variants were annotated using Ensembl’s Variant Effect Predictor (VEP) release 103.1.(4) The *LDLR* variants in the canonical transcript ENST00000558518 were filtered for a minor allele frequency (MAF) of 0.0006, which is equal to the frequency of the most common FH variant (p.Arg3527Gln in *APOB*) according to the gnomAD database version 2.1.1.(5) Further variant filtering steps included a minimum read depth of 10 and genotype quality of 20. The SAMtools plugin split-vep was used to keep variants that had a predicted consequence of missense or worse, and the resulting variants with a SIFT annotation of “tolerated” or a PolyPhen annotation of “benign” were excluded.(3,6,7) These filtering steps were followed by manual curation of the variants by two expert reviewers (M.F. and S.E.H.) who respected the Association for Clinical Genomics Science (ACGS) guidelines and the evidence accrued from the LOVD database for *LDLR*.(8,9) For the *APOE* gene, the heterozygous p.Leu167del in-frame deletion was considered to be FH-causing, and the pathogenic variants in *PCSK9* and *APOB* were filtered based on a pre-defined list of curated variants with functional assay backing.(10,11)

**CHD and CVD definition codes**

CHD was defined with the International Classification of Diseases 10 (ICD-10) codes I21, I22, I23, I24, I25.0, I25.1, I25.2, I25.3, I25.5, 125.6, I25.8, 125.9, I48, I50, I11.0, I13.0, I13.2, I32.2, I61, I63 and the Office of Population Censuses and Surveys Classification of Interventions and Procedures 4 (OPCS-4) codes K40, K41, K42, K43, K44.1, K44.8, K44.9, K45.1, K45.2, K45.3, K45.4, K45.5, K45.8, K45.9, K46.1, K46.2, K46.3, K46.4, K46.8, K46.9, K47.1, K49, K50, K75, K52.1, K57.1, K57.5, K62.1, K62.2, K62.3, K62.4, K62.5, X50.1, X50.2. CVD was defined with the ICD-10 codes I21, I22, I23, I24, I25.0, I25.1, I25.2, I25.3, I25.5, 125.6, I25.8, 125.9 and the OPCS-4 codes K40, K41, K42, K43, K44.1, K44.8, K44.9, K45.1, K45.2, K45.3, K45.4, K45.5, K45.8, K45.9, K46.1, K46.2, K46.3, K46.4, K46.8, K46.9, K47.1, K49, K50, K75.

**Software**

The data analysis and figures were done in R version 4.0.2.(12) The R package tableone version 0.12.0. was used to make Table 1.(13) The receiver operating characteristic (ROC) curves were plotted with the R package pROC version 1.16.2.(14) The NRI analysis was done using the R package nricens version 1.6.(15)

**References**

1. gavinband / bgen / wiki / bgenix — Bitbucket. Available at: https://bitbucket.org/gavinband/bgen/wiki/bgenix. (Accessed: 20th March 2020)

2. Mitchell, R. E. et al. UK Biobank Genetic Data: MRC-IEU Quality Control, version 2, 18/01/2019.

3. Danecek, P. et al. Twelve years of SAMtools and BCFtools. Gigascience 10, (2021).

4. McLaren, W. et al. The Ensembl Variant Effect Predictor. Genome Biol. 2016 171 17, 1–14 (2016).

5. Karczewski, K. J. et al. The mutational constraint spectrum quantified from variation in 141,456 humans. Nat. 2020 5817809 581, 434–443 (2020).

6. Adzhubei, I. A. et al. A method and server for predicting damaging missense mutations. Nat. Methods 2010 74 7, 248–249 (2010).

7. Kumar, P., Henikoff, S. & Ng, P. C. Predicting the effects of coding non-synonymous variants on protein function using the SIFT algorithm. (2009). doi:10.1038/nprot.2009.86

8. Ellard, S. et al. ACGS Best Practice Guidelines for Variant Classification in Rare Disease 2020 Recommendations ratified by ACGS Quality Subcommittee on 4 th. (2020). doi:10.1101/531210

9. Leigh, S. et al. The UCL low-density lipoprotein receptor gene variant database: pathogenicity update. J. Med. Genet. 54, 217–223 (2017).

10. Awan, Z. et al. APOE p.Leu167del mutation in familial hypercholesterolemia. Atherosclerosis 231, 218–222 (2013).

11. Lázaro, C., Lerner-Ellis, J. & Spurdle, A. Clinical DNA variant interpretation theory and practice. (Academic Press, 2021).

12. R: The R Project for Statistical Computing. Available at: https://www.r-project.org/. (Accessed: 11th February 2022)

13. Panos, A. & Mavridis, D. TableOne: an online web application and R package for summarising and visualising data. Evid. Based. Ment. Health 23, 127–130 (2020).

14. Robin, X. et al. pROC: An open-source package for R and S+ to analyze and compare ROC curves. BMC Bioinformatics 12, 1–8 (2011).

15. Inoue, E. nricens: NRI for Risk Prediction Models with Time to Event and Binary Response Data. (2018).

**SUPPLEMENTL RESULTS**

**LDL-C polygenic score (PGS)**

The p-value cut-off of 5x10^-4^ for the variants of GLGC’s LDL-C genome-wide association study (GWAS) summary statistics included 10,137 variants. After running the LASSO regression on these variants using the LASSO training dataset described in the methods section, 1,466 genetic variants were retained by the model. The LDL-C PGS r-squared was 0.14 (95% confidence intervals (CI): 0.13-0.15) in the independent test data.

**Model performance across age groups**

To ensure that the LDL-C adjusted for statin use model was not underperforming in older individuals compared to younger individuals, we performed an age-stratified analysis. The AUC in the test data for the 40-50 age band was 0.76 (95% CI: 0.0.63; 0.88), for the 50-60 age band was 0.68 (95% CI: 0.57; 0.78), and for the 60+ age band was 0.72 (95% CI: 0.64; 0.81). When performing an interaction test between the 40-50 age band compared to the other age bands, we obtain non-significant group differences: p-value = 0.34 for 40-50 versus 50-60 age band, and p-value = 0.60 for 40-50 versus 60+ age band. We also performed this interaction test for the multivariable model containing the LDL-C PGS and obtained non-significant group differences: p-value = 0.29 for 40-50 versus 50-60 age band, and p-value = 0.80 for 40-60 versus 60+ age band. This indicates that the models did not perform significantly differently in these difference age categories.

**Supplemental Table 1. Genetic coordinates of FH-causing genes.** Genetic coordinates are mapped to GRCh38.

| **Gene name** | **Chromosome number** | **Start coordinate** | **End coordinate** |
| --- | --- | --- | --- |
| *LDLR* | 19 | 11,089,262 | 11,133,820 |
| *APOB* | 2 | 21,001,429 | 21,044,073 |
| *APOE* | 19 | 44,905,791 | 44,909,393 |
| *PCSK9* | 1 | 55,039,347 | 55,064,852 |

**Supplemental Table 2. Autosomal dominant FH-causing mutation identified in our study cohort.** Genetic coordinates are mapped to GRCh38.

| **Gene** | **Chromosome** | **Position** | **Reference allele** | **Alternate allele** | **Nucleotide change** | **Protein** | **Number of carriers** | **UKB frequency (1/n)** |
| --- | --- | --- | --- | --- | --- | --- | --- | --- |
| *APOB* | 2 | 21006289 | G | A | c.10579C>T | p.Arg3527Trp | 2 | 70,220 |
|  |  | 21006288 | C | T | c.10580G>A | p.Arg3527Gln | 99 | 1,419 |
| *APOE* | 19 | 44908791 | GCTC | G | c.499_501del | p.Leu167del | 13 | 10,803 |
| *LDLR* | 19 | 11100236 | C | G | c.81C>G | p.Cys27Trp | 1 | 140,439 |
|  |  | 11100291 | T | G | c.136T>G | p.Cys46Gly | 1 | 140,439 |
|  |  | 11100294 | G | A | c.139G>A | p.Asp47Asn | 5 | 28,088 |
|  |  | 11102705 | C | T | c.232C>T | p.Arg78Cys | 13 | 10,803 |
|  |  | 11102714 | C | T | c.241C>T | p.Arg81Cys | 2 | 70,220 |
|  |  | 11102732 | T | G | c.259T>G | p.Trp87Gly | 6 | 23,407 |
|  |  | 11102741 | G | A | c.268G>A | p.Asp90Asn | 5 | 28,088 |
|  |  | 11102765 | G | A | c.292G>A | p.Gly98Ser | 10 | 14,044 |
|  |  | 11102774 | G | A | c.301G>A | p.Glu101Lys | 12 | 11,703 |
|  |  | 11102787 | G | A | c.313+1G>A | . | 5 | 28,088 |
|  |  | 11102787 | G | C | c.313+1G>C | . | 1 | 140,439 |
|  |  | 11102787 | G | GT | c.313+2dup | . | 2 | 70,220 |
|  |  | 11105249 | C | T | c.343C>T | p.Arg115Cys | 2 | 70,220 |
|  |  | 11105268 | G | T | c.362G>T | p.Cys121Phe | 2 | 70,220 |
|  |  | 11105324 | G | A | c.418G>A | p.Glu140Lys | 1 | 140,439 |
|  |  | 11105339 | GTGCTCACCTGTGGTCCCGCCAGC | G | c.435_457del | p.Leu146ProfsTer26 | 1 | 140,439 |
|  |  | 11105407 | C | A | c.501C>A | p.Cys167Ter | 2 | 70,220 |
|  |  | 11105408 | G | A | c.502G>A | p.Asp168Asn | 14 | 10,031 |
|  |  | 11105415 | AC | A | c.513del | p.Asp172ThrfsTer34 | 1 | 140,439 |
|  |  | 11105448 | C | G | c.542C>G | p.Pro181Arg | 2 | 70,220 |
|  |  | 11105549 | C | T | c.643C>T | p.Arg215Cys | 4 | 35,110 |
|  |  | 11105567 | G | A | c.661G>A | p.Asp221Asn | 2 | 70,220 |
|  |  | 11105568 | A | G | c.662A>G | p.Asp221Gly | 5 | 28,088 |
|  |  | 11105585 | GAC | G | c.680_681del | p.Asp227GlyfsTer12 | 4 | 35,110 |
|  |  | 11105585 | GAC | GAG | c.681delinsG | p.Asp227Glu | 2 | 70,220 |
|  |  | 11105588 | G | T | c.682G>T | p.Glu228Ter | 2 | 70,220 |
|  |  | 11105589 | AG | A | c.685del | p.Glu229LysfsTer36 | 1 | 140,439 |
|  |  | 11106579 | C | T | c.709C>T | p.Arg237Cys | 1 | 140,439 |
|  |  | 11106588 | G | A | c.718G>A | p.Glu240Lys | 20 | 7,022 |
|  |  | 11106592 | T | C | c.722T>C | p.Phe241Ser | 1 | 140,439 |
|  |  | 11106631 | A | C | c.761A>C | p.Gln254Pro | 1 | 140,439 |
|  |  | 11107432 | C | A | c.858C>A | p.Ser286Arg | 1 | 140,439 |
|  |  | 11107433 | G | A | c.859G>A | p.Gly287Ser | 4 | 35,110 |
|  |  | 11107436 | G | A | c.862G>A | p.Glu288Lys | 1 | 140,439 |
|  |  | 11107461 | G | A | c.887G>A | p.Cys296Tyr | 1 | 140,439 |
|  |  | 11107481 | C | T | c.907C>T | p.Arg303Trp | 2 | 70,220 |
|  |  | 11107486 | C | G | c.912C>G | p.Asp304Glu | 4 | 35,110 |
|  |  | 11107512 | G | A | c.938G>A | p.Cys313Tyr | 2 | 70,220 |
|  |  | 11110660 | G | A | c.949G>A | p.Glu317Lys | 35 | 4,013 |
|  |  | 11110678 | G | A | c.967G>A | p.Gly323Ser | 1 | 140,439 |
|  |  | 11110714 | G | A | c.1003G>A | p.Gly335Ser | 3 | 46,813 |
|  |  | 11110738 | G | A | c.1027G>A | p.Gly343Ser | 8 | 17,555 |
|  |  | 11110759 | C | T | c.1048C>T | p.Arg350Ter | 4 | 35,110 |
|  |  | 11110760 | G | C | c.1049G>C | p.Arg350Pro | 4 | 35,110 |
|  |  | 11111571 | G | A | c.1118G>A | p.Gly373Asp | 1 | 140,439 |
|  |  | 11111619 | C | T | c.1166C>T | p.Thr389Met | 8 | 17,555 |
|  |  | 11113286 | G | A | c.1195G>A | p.Ala399Thr | 1 | 140,439 |
|  |  | 11113287 | C | A | c.1196C>A | p.Ala399Asp | 1 | 140,439 |
|  |  | 11113292 | CTCTTC | CTCT | c.1205_1206del | p.Phe403HisfsTer37 | 1 | 140,439 |
|  |  | 11113307 | C | T | c.1216C>T | p.Arg406Trp | 5 | 28,088 |
|  |  | 11113308 | G | A | c.1217G>A | p.Arg406Gln | 4 | 35,110 |
|  |  | 11113313 | G | A | c.1222G>A | p.Glu408Lys | 1 | 140,439 |
|  |  | 11113322 | A | G | c.1231A>G | p.Lys411Glu | 1 | 140,439 |
|  |  | 11113329 | C | T | c.1238C>T | p.Thr413Met | 14 | 10,031 |
|  |  | 11113337 | C | T | c.1246C>T | p.Arg416Trp | 2 | 70,220 |
|  |  | 11113419 | G | C | c.1328G>C | p.Trp443Ser | 1 | 140,439 |
|  |  | 11113426 | C | G | c.1335C>G | p.Asp445Glu | 5 | 28,088 |
|  |  | 11113554 | CA | C | c.1379del | p.His460ProfsTer47 | 1 | 140,439 |
|  |  | 11113590 | G | T | c.1414G>T | p.Asp472Tyr | 6 | 23,407 |
|  |  | 11113608 | G | A | c.1432G>A | p.Gly478Arg | 2 | 70,220 |
|  |  | 11113612 | T | C | c.1436T>C | p.Leu479Pro | 2 | 70,220 |
|  |  | 11113620 | G | A | c.1444G>A | p.Asp482Asn | 29 | 4,843 |
|  |  | 11113650 | G | A | c.1474G>A | p.Asp492Asn | 1 | 140,439 |
|  |  | 11113678 | C | T | c.1502C>T | p.Ala501Val | 5 | 28,088 |
|  |  | 11113705 | C | T | c.1529C>T | p.Thr510Met | 3 | 46,813 |
|  |  | 11113743 | G | A | c.1567G>A | p.Val523Met | 1 | 140,439 |
|  |  | 11116095 | T | G | c.1588T>G | p.Phe530Val | 10 | 14,044 |
|  |  | 11116125 | G | A | c.1618G>A | p.Ala540Thr | 2 | 70,220 |
|  |  | 11116141 | G | A | c.1634G>A | p.Gly545Glu | 1 | 140,439 |
|  |  | 11116198 | A | G | c.1691A>G | p.Asn564Ser | 2 | 70,220 |
|  |  | 11116873 | C | T | c.1720C>T | p.Arg574Cys | 2 | 70,220 |
|  |  | 11116898 | T | C | c.1745T>C | p.Leu582Pro | 1 | 140,439 |
|  |  | 11116918 | G | A | c.1765G>A | p.Asp589Asn | 1 | 140,439 |
|  |  | 11116928 | G | A | c.1775G>A | p.Gly592Glu | 1 | 140,439 |
|  |  | 11116936 | C | T | c.1783C>T | p.Arg595Trp | 6 | 23,407 |
|  |  | 11116937 | G | A | c.1784G>A | p.Arg595Gln | 2 | 70,220 |
|  |  | 11116976 | C | G | c.1823C>G | p.Pro608Arg | 1 | 140,439 |
|  |  | 11120091 | G | A | c.1846-1G>A | . | 1 | 140,439 |
|  |  | 11120106 | G | T | c.1860G>T | p.Trp620Cys | 1 | 140,439 |
|  |  | 11120110 | GAT | G | c.1867_1868del | p.Ile623HisfsTer21 | 1 | 140,439 |
|  |  | 11120143 | C | T | c.1897C>T | p.Arg633Cys | 9 | 15,604 |
|  |  | 11120144 | G | A | c.1898G>A | p.Arg633His | 1 | 140,439 |
|  |  | 11120152 | G | A | c.1906G>A | p.Gly636Ser | 3 | 46,813 |
|  |  | 11120212 | C | A | c.1966C>A | p.His656Asn | 8 | 17,555 |
|  |  | 11120370 | G | A | c.1988G>A | p.Gly663Glu | 1 | 140,439 |
|  |  | 11120408 | G | A | c.2026G>A | p.Gly676Ser | 5 | 28,088 |
|  |  | 11120436 | C | T | c.2054C>T | p.Pro685Leu | 12 | 11,703 |
|  |  | 11120441 | A | T | c.2059A>T | p.Ile687Phe | 5 | 28,088 |
|  |  | 11120442 | T | TC | c.2061dup | p.Asn688GlnfsTer29 | 1 | 140,439 |
|  |  | 11123200 | G | T | c.2167G>T | p.Glu723Ter | 1 | 140,439 |
|  |  | 11128027 | C | CA | c.2332dup | p.Arg778LysfsTer4 | 1 | 140,439 |

**Supplemental Table 3. List of variants of unknown significance (VUS) excluded from the analysis.** Genetic coordinates are mapped to GRCh38. Count refers to the number of participants having the VUS.

| **Gene** | **Chromosome number** | **Position** | **Reference allele** | **Alternate allele** | **HGVSc** | **HGVSp** | **Count** |
| --- | --- | --- | --- | --- | --- | --- | --- |
| *APOB* | 2 | 21001939 | ACTG | A | ENST00000233242:c.13480_13482delCAG | ENSP00000233242.1:p.Gln4494del | 132 |
|  |  | 21006196 | C | T | ENST00000233242:c.10672C>T | ENSP00000233242.1:p.Arg3558Cys | 299 |
|  |  | 21006239 | C | G | ENST00000233242:c.10629C>G | ENSP00000233242.1:p.Asn3543Lys | 3 |
|  |  | 21006349 | C | T | ENST00000233242:c.10519C>T | ENSP00000233242.1:p.Arg3507Trp | 1 |
|  |  | 21015387 | G | C | ENST00000233242:c.3491G>C | ENSP00000233242.1:p.Arg1164Thr | 1 |
| *PCSK9* | 1 | 55044021 | A | G | ENST00000302118:c.386A>G | ENSP00000303208.5:p.Asp129Gly | 2 |
|  |  | 55052698 | G | A | ENST00000302118:c.706G>A | ENSP00000303208.5:p.Gly236Ser | 4 |
|  |  | 55058543 | C | G | ENST00000302118:c.1399C>G | ENSP00000303208.5:p.Pro467Ala | 3 |
| *LDLR* | 19 | 11100261 | G | C | ENST00000558518.6:c.106G>C | ENSP00000454071.1:p.Asp36His | 1 |
|  |  | 11100322 | C | T | ENST00000558518.6:c.167C>T | ENSP00000454071.1:p.Ser56Phe | 1 |
|  |  | 11100328 | A | T | ENST00000558518.6:c.173A>T | ENSP00000454071.1:p.Glu58Val | 2 |
|  |  | 11100340 | C | T | ENST00000558518.6:c.185C>T | ENSP00000454071.1:p.Thr62Met | 10 |
|  |  | 11102720 | A | T | ENST00000558518.6:c.247A>T | ENSP00000454071.1:p.Ile83Phe | 1 |
|  |  | 11105262 | G | C | ENST00000558518.6:c.356G>C | ENSP00000454071.1:p.Gly119Ala | 1 |
|  |  | 11105337 | C | T | ENST00000558518.6:c.431C>T | ENSP00000454071.1:p.Pro144Leu | 1 |
|  |  | 11105379 | C | T | ENST00000558518.6:c.473C>T | ENSP00000454071.1:p.Ser158Phe | 1 |
|  |  | 11105414 | G | A | ENST00000558518.6:c.508G>A | ENSP00000454071.1:p.Asp170Asn | 22 |
|  |  | 11105415 | AC | GC | ENST00000558518.6:c.509delinsG | ENSP00000454071.1:p.Asp170Gly | 1 |
|  |  | 11106580 | G | A | ENST00000558518.6:c.710G>A | ENSP00000454071.1:p.Arg237His | 10 |
|  |  | 11106593 | C | A | ENST00000558518.6:c.723C>A | ENSP00000454071.1:p.Phe241Leu | 3 |
|  |  | 11106601 | C | G | ENST00000558518.6:c.731C>G | ENSP00000454071.1:p.Ser244Cys | 1 |
|  |  | 11106639 | C | T | ENST00000558518.6:c.769C>T | ENSP00000454071.1:p.Arg257Trp | 2 |
|  |  | 11107472 | A | G | ENST00000558518.6:c.898A>G | ENSP00000454071.1:p.Arg300Gly | 1 |
|  |  | 11111538 | A | C | ENST00000558518.6:c.1085A>C | ENSP00000454071.1:p.Asp362Ala | 60 |
|  |  | 11111558 | G | A | ENST00000558518.6:c.1105G>A | ENSP00000454071.1:p.Val369Met | 3 |
|  |  | 11111609 | G | T | ENST00000558518.6:c.1156G>T | ENSP00000454071.1:p.Asp386Tyr | 5 |
|  |  | 11113278 | G | T | ENST00000558518.6:c.1187G>T | ENSP00000454071.1:p.Gly396Val | 1 |
|  |  | 11113287 | C | T | ENST00000558518.6:c.1196C>T | ENSP00000454071.1:p.Ala399Val | 1 |
|  |  | 11113292 | CTCTTC | CTCTTG | ENST00000558518.6:c.1206delinsG | ENSP00000454071.1:p.Phe402Leu | 1 |
|  |  | 11113362 | C | T | ENST00000558518.6:c.1271C>T | ENSP00000454071.1:p.Pro424Leu | 3 |
|  |  | 11113374 | A | C | ENST00000558518.6:c.1283A>C | ENSP00000454071.1:p.Asn428Thr | 1 |
|  |  | 11113409 | A | G | ENST00000558518.6:c.1318A>G | ENSP00000454071.1:p.Arg440Gly | 4 |
|  |  | 11113561 | TCTCTTCCTA | TCTCTTACTA | ENST00000558518.6:c.1391delinsA | ENSP00000454071.1:p.Ser464Tyr | 2 |
|  |  | 11113625 | G | T | ENST00000558518.6:c.1449G>T | ENSP00000454071.1:p.Trp483Cys | 1 |
|  |  | 11113751 | T | G | ENST00000558518.6:c.1575T>G | ENSP00000454071.1:p.Asp525Glu | 6 |
|  |  | 11113762 | G | T | ENST00000558518.6:c.1586G>T | ENSP00000454071.1:p.Gly529Val | 1 |
|  |  | 11116101 | T | C | ENST00000558518.6:c.1594T>C | ENSP00000454071.1:p.Tyr532His | 1 |
|  |  | 11116132 | T | A | ENST00000558518.6:c.1625T>A | ENSP00000454071.1:p.Ile542Asn | 1 |
|  |  | 11116205 | C | G | ENST00000558518.6:c.1698C>G | ENSP00000454071.1:p.Ile566Met | 1 |
|  |  | 11116885 | G | A | ENST00000558518.6:c.1732G>A | ENSP00000454071.1:p.Val578Ile | 2 |
|  |  | 11116914 | C | G | ENST00000558518.6:c.1761C>G | ENSP00000454071.1:p.Ser587Arg | 4 |
|  |  | 11116949 | T | C | ENST00000558518.6:c.1796T>C | ENSP00000454071.1:p.Leu599Ser | 4 |
|  |  | 11116970 | C | A | ENST00000558518.6:c.1817C>A | ENSP00000454071.1:p.Ala606Asp | 14 |
|  |  | 11120454 | C | T | ENST00000558518.6:c.2072C>T | ENSP00000454071.1:p.Ser691Leu | 4 |
|  |  | 11120484 | G | T | ENST00000558518.6:c.2102G>T | ENSP00000454071.1:p.Gly701Val | 1 |
|  |  | 11120507 | A | G | ENST00000558518.6:c.2125A>G | ENSP00000454071.1:p.Arg709Gly | 1 |
|  |  | 11123315 | C | T | ENST00000558518.6:c.2282C>T | ENSP00000454071.1:p.Thr761Met | 11 |
|  |  | 11128062 | C | A | ENST00000558518.6:c.2366C>A | ENSP00000454071.1:p.Ala789Asp | 1 |
|  |  | 11129553 | G | C | ENST00000558518.6:c.2430G>C | ENSP00000454071.1:p.Trp810Cys | 1 |
|  |  | 11129573 | A | T | ENST00000558518.6:c.2450A>T | ENSP00000454071.1:p.Asn817Ile | 1 |
|  |  | 11129582 | G | A | ENST00000558518.6:c.2459G>A | ENSP00000454071.1:p.Ser820Asn | 1 |
|  |  | 11129633 | A | G | ENST00000558518.6:c.2510A>G | ENSP00000454071.1:p.His837Arg | 18 |
|  |  | 11129653 | G | A | ENST00000558518.6:c.2530G>A | ENSP00000454071.1:p.Gly844Ser | 1 |
|  |  | 11131299 | G | C | ENST00000558518.6:c.2566G>C | ENSP00000454071.1:p.Glu856Gln | 1 |

**Supplemental Table 4. UK Biobank participant characteristics post imputation of missing values stratified by familial hypercholesterolaemia (FH) carriership.** The p-values shown in the table are from the Kruskal-Wallis Rank Sum test for continuous variables, and from the Man-Whitney U test for binary variables. BMI = body mass index; CHD = coronary heart disease; CVD = cardiovascular disease; HDL-C = high-density lipoprotein cholesterol; IQR = interquartile range; LDL-C = low-density lipoprotein cholesterol; PGS = polygenic score.

|  | **Mutation negative** | **Mutation positive** | **p-value of differences** |
| --- | --- | --- | --- |
| n | 139291 | 488 |  |
| Sex (male) (%) | 63382 (45.5) | 207 (42.4) | 0.187 |
| Age (median [IQR]) | 58.0 [51.0, 63.0] | 58.0 [51.0, 63.0] | 0.803 |
| Townsend deprivation index (median [IQR]) | -2.4 [-3.8, 0.0] | -2.2 [-3.7, 0.1] | 0.367 |
| BMI, kg/m2 (median [IQR]) | 26.7 [24.1, 29.8] | 27.1 [23.9, 29.8] | 0.647 |
| Smoking status (%) |  |  | 0.827 |
| Non-smoker | 79618 (57.2) | 281 (57.6) |  |
| Former smoker | 51177 (36.7) | 173 (35.5) |  |
| Light smoker (<10 cigarettes/day) | 2021 (1.5) | 7 (1.4) |  |
| Moderate smoker (10-19 cigarettes/day) | 3497 (2.5) | 13 (2.7) |  |
| Heavy Smoker (>20 cigarettes/day) | 2978 (2.1) | 14 (2.9) |  |
| Alcohol consumption (%) |  |  | 0.492 |
| Prefer not to answer | 88 (0.1) | 1 (0.2) |  |
| 1/day | 29719 (21.3) | 93 (19.1) |  |
| 3-4 times/week | 34015 (24.4) | 135 (27.7) |  |
| 1-2 times/week | 36823 (26.4) | 130 (26.6) |  |
| 1-3 times/month | 15498 (11.1) | 54 (11.1) |  |
| Special occasions | 14383 (10.3) | 45 (9.2) |  |
| Never | 8765 (6.3) | 30 (6.1) |  |
| Family history of CHD (%) | 67013 (48.1) | 306 (62.7) | <0.001 |
| Systolic blood pressure, mmHg (median [IQR]) | 136.5 [125.0, 149.5] | 135.0 [124.5, 148.5] | 0.109 |
| Diastolic blood pressure, mmHg (median [IQR]) | 82.0 [75.0, 89.0] | 81.0 [74.0, 87.0] | 0.024 |
| Hypertension (%) | 7946 (5.7) | 35 (7.2) | 0.195 |
| Statin use (%) | 18139 (13.0) | 165 (33.8) | <0.001 |
| LDL-C PGS, mmol/L (median [IQR]) | 3.7 [3.5, 3.9] | 3.7 [3.5, 3.9] | 0.652 |
| **Blood biomarkers** |  |  |  |
| LDL-C (unadjusted for statin use), mmol/L (median [IQR]) | 3.5 [3.0, 4.1] | 3.9 [3.2, 4.8] | <0.001 |
| LDL-C (adjusted for statin use), mmol/L (median [IQR]) | 3.7 [3.1, 4.2] | 4.4 [3.7, 5.4] | <0.001 |
| HDL-C, mmol/L (median [IQR]) | 1.4 [1.2, 1.7] | 1.4 [1.2, 1.7] | 0.199 |
| Total cholesterol, mmol/L (median [IQR]) | 5.7 [4.9, 6.4] | 6.0 [5.1, 7.2] | <0.001 |
| Lipoprotein(a), nmol/L (median [IQR]) | 17.9 [9.8, 55.3] | 21.3 [12.3, 53.1] | 0.223 |
| Apolipoprotein A1, g/L (median [IQR]) | 1.5 [1.4, 1.7] | 1.5 [1.3, 1.7] | <0.001 |
| Apolipoprotein B, g/L (median [IQR]) | 1.0 [0.9, 1.2] | 1.1 [1.0, 1.4] | <0.001 |
| Triglycerides, mmol/L (median [IQR]) | 1.5 [1.1, 2.2] | 1.3 [0.9, 1.9] | <0.001 |
| C-reactive protein, mg/L (median [IQR]) | 1.3 [0.7, 2.7] | 1.2 [0.6, 2.4] | 0.045 |
| Aspartate aminotransferase, um (median [IQR]) | 24.4 [21.0, 28.8] | 25.2 [21.0, 29.5] | 0.089 |
| Alanine aminotransferase, um (median [IQR]) | 20.1 [15.4, 27.3] | 20.2 [15.6, 27.4] | 0.830 |
| Alkaline phosphatase, um (median [IQR]) | 80.1 [67.1, 95.5] | 80.6 [66.5, 95.8] | 0.571 |
| **Disease prevalence & incidence** |  |  |  |
| CHD prevalence (%) | 3890 (2.8) | 40 (8.2) | <0.001 |
| CHD incidence (%) | 5370 (3.9) | 32 (6.6) | 0.003 |
| CVD prevalence (%) | 5686 (4.1) | 45 (9.2) | <0.001 |
| CVD incidence (%) | 9038 (6.5) | 46 (9.4) | 0.011 |
| Type 2 diabetes prevalence (%) | 3593 (2.6) | 11 (2.3) | 0.757 |
| Type 2 diabetes incidence (%) | 4948 (3.6) | 19 (3.9) | 0.776 |

**Supplemental Table 5. UK Biobank variables in LASSO models post imputation of missing values stratified by testing vs training dataset.** The p-values shown in the table are from the Kruskal-Wallis Rank Sum test for continuous variables, and from the Man-Whitney U test for binary variables. BMI = body mass index; CHD = coronary heart disease; HDL-C = high-density lipoprotein cholesterol; IQR = interquartile range; LDL-C = low-density lipoprotein cholesterol; PGS = polygenic score

|  | **Testing data** | **Training data** | **P-value of differences** |
| --- | --- | --- | --- |
| n | 27955 | 111824 |  |
| Age (median [IQR]) | 58.00 [51.00, 63.00] | 58.00 [51.00, 63.00] | 0.576 |
| Sex (male) (%) | 12704 (45.4) | 50885 (45.5) | 0.862 |
| Townsend deprivation index (median [IQR]) | -2.39 [-3.76, 0.00] | -2.37 [-3.76, 0.02] | 0.288 |
| Alcohol consumption (%) |  |  | 0.114 |
| Prefer not to answer | 20 (0.1) | 69 (0.1) |  |
| 1/day | 5981 (21.4) | 23831 (21.3) |  |
| 3-4 times/week | 6856 (24.5) | 27294 (24.4) |  |
| 1-2 times/week | 7500 (26.8) | 29453 (26.3) |  |
| 1-3 times/month | 3043 (10.9) | 12509 (11.2) |  |
| Special occasions | 2773 (9.9) | 11655 (10.4) |  |
| Never | 1782 (6.4) | 7013 (6.3) |  |
| Smoking status (%) |  |  | 0.636 |
| Non-smoker | 16077 (57.5) | 63822 (57.1) |  |
| Former smoker | 10185 (36.4) | 41165 (36.8) |  |
| Light smoker (<10 cigarettes/day) | 408 (1.5) | 1620 (1.4) |  |
| Moderate smoker (10-19 cigarettes/day) | 707 (2.5) | 2803 (2.5) |  |
| Heavy Smoker (>20 cigarettes/day) | 578 (2.1) | 2414 (2.2) |  |
| Family history of CHD (%) | 13450 (48.1) | 53869 (48.2) | 0.863 |
| BMI, kg/m2 (median [IQR]) | 26.64 [24.09, 29.76] | 26.68 [24.10, 29.79] | 0.324 |
| Systolic blood pressure, mmHg (median [IQR]) | 136.50 [125.00, 149.50] | 136.50 [125.00, 149.50] | 0.835 |
| Diastolic blood pressure, mmHg (median [IQR]) | 82.00 [75.50, 89.00] | 82.00 [75.00, 89.00] | 0.684 |
| Hypertension (%) | 1601 (5.7) | 6380 (5.7) | 0.900 |
| Statin use (%) | 3674 (13.1) | 14630 (13.1) | 0.800 |
| FH mutation carrier (%) | 93 (0.3) | 395 (0.4) | 0.642 |
| Type 2 diabetes prevalence (%) | 689 (2.5) | 2915 (2.6) | 0.187 |
| LDL-C PGS (median [IQR]) | 3.73 [3.52, 3.92] | 3.73 [3.52, 3.92] | 0.681 |
| **Blood biomarkers** |  |  |  |
| LDL-C, mmol/L (median [IQR]) | 3.54 [2.97, 4.13] | 3.53 [2.96, 4.13] | 0.510 |
| HDL-C, mmol/L (median [IQR]) | 1.42 [1.18, 1.70] | 1.41 [1.18, 1.70] | 0.119 |
| Total cholesterol, mmol/L (median [IQR]) | 5.68 [4.95, 6.44] | 5.68 [4.94, 6.45] | 0.459 |
| Triglycerides, mmol/L (median [IQR]) | 1.48 [1.04, 2.13] | 1.49 [1.05, 2.15] | 0.135 |
| Lipoprotein(a), nmol/L (median [IQR]) | 17.72 [9.83, 55.05] | 17.90 [9.76, 55.30] | 0.728 |
| Apolipoprotein A, g/L (median [IQR]) | 1.52 [1.36, 1.71] | 1.52 [1.36, 1.71] | 0.106 |
| Apolipoprotein B, g/L (median [IQR]) | 1.02 [0.87, 1.18] | 1.02 [0.87, 1.18] | 0.943 |
| C-reactive protein, mg/L (median [IQR]) | 1.29 [0.64, 2.66] | 1.31 [0.65, 2.70] | 0.073 |
| Aspartate aminotransferase, um (median [IQR]) | 24.30 [21.00, 28.70] | 24.40 [21.00, 28.80] | 0.123 |
| Alanine aminotransferase, um (median [IQR]) | 20.12 [15.43, 27.21] | 20.11 [15.42, 27.29] | 0.422 |
| Alkaline phosphatase, um (median [IQR]) | 80.10 [66.90, 95.50] | 80.10 [67.20, 95.40] | 0.509 |

**Supplemental Table 6.** **The non-genetic variables and coefficients retained by LASSO regression for monogenic FH prediction.** The c-statistic of the independent test dataset was equal to 0.76 (95% CI: 0.71; 0.82). The variables were standardised prior to running the LASSO regression: the mean and SD are given in the table. Apo-A1 = apolipoprotein A1; CHD = coronary heart disease; FH = familial hypercholesterolaemia; LDL-C = low-density lipoprotein cholesterol; PGS = polygenic score; SD = standard deviation.

|  | **Coefficients** | **Mean** | **SD** |
| --- | --- | --- | --- |
| **(Intercept)** | -6.014496 |  |  |
| **Age** | -0.071679 | 56.86088 | 7.971519 |
| **Statin use** | 0.180699 | 0.1314255 | 0.3378712 |
| **Systolic blood pressure** | -0.005447 | 138.0973 | 18.44399 |
| **Diastolic blood pressure** | -0.060420 | 82.26036 | 10.08779 |
| **Apo-A1** | -0.258628 | 1.552397 | 0.2721509 |
| **Triglycerides** | -0.561805 | 1.738264 | 1.009821 |
| **Family history of CHD** | 0.146255 | 0.4811304 | 0.4996527 |
| **LDL-C x LDL-C** | 0.626778 | 13.53496 | 6.539555 |
| **Statin use x LDL-C** | 0.406210 | 0.3680052 | 0.9784316 |

**Supplemental Table 7. The variables and coefficients retained by LASSO regression for monogenic FH prediction including LDL-C PGS.** The variables were standardised prior to running the LASSO regression: the mean and SD are given in the table. ALT = Alanine aminotransferase; Apo-A1 = apolipoprotein A1; BMI = body mass index; CHD = coronary heart disease; CRP = C-reactive protein; HDL-C = high-density lipoprotein cholesterol; LDL-C = low-density lipoprotein cholesterol; PGS = polygenic score; SD = standard deviation.

|  | **Coefficients** | **Mean** | **SD** |
| --- | --- | --- | --- |
| **(Intercept)** | -6.061379 |  |  |
| **LDL-C** | 0.252485 | 3.575719 | 0.8655747 |
| **BMI** | -0.005819 | 27.30579 | 4.653879 |
| **Statin use** | 0.381582 | 0.1314255 | 0.3378712 |
| **Diastolic blood pressure** | -0.082472 | 82.26036 | 10.08779 |
| **Apo-A1** | -0.282415 | 1.552397 | 0.2721509 |
| **Triglycerides** | -0.600269 | 1.738264 | 1.009821 |
| **CRP** | -0.004564 | 2.521555 | 4.347809 |
| **ALT** | -0.017213 | 23.38488 | 13.94569 |
| **LDL-C PGS** | -0.190587 | 3.706099 | 0.3052653 |
| **Family history of CHD** | 0.161401 | 0.4811304 | 0.4996527 |
| **Prevalent type 2 diabetes** | -0.002958 | 0.02464675 | 0.1550489 |
| **Age x LDL-C PGS** | -0.169897 | 210.7329 | 34.35274 |
| **LDL-C x LDL-C** | 0.520575 | 13.53496 | 6.539555 |
| **Statin use x LDL-C** | 0.314738 | 0.3680052 | 0.9784316 |

**Supplemental Table 8. The classification accuracy of an algorithm for predicting monogenic familial hypercholesterolaemia (FH) using the multivariable model and LDL-C concentration accounting for statin use.** There are 93 FH-causing variant positive participants in the test data comprising of a total of 27,955 participants.

| **Predicted probability cut-off** | % sensitivity (95%CI) | % false positive rate (95%CI) | % positive predictive value (95%CI) | % negative predictive value (95%CI) | FH-causing variants below threshold | FH-causing variants above threshold | Controls above threshold |
| --- | --- | --- | --- | --- | --- | --- | --- |
| **Multivariable model** | | | | | | | |
| **0.05** | 7.5 (3.7;14.7) | 0.2 (0.1;0.2) | 13.0 (6.4;24.4) | 99.7 (99.6;99.8) | 86 | 7 | 47 |
| **0.02** | 20.4 (13.5;29.7) | 1.1 (0.9;1.2) | 6.0 (3.9;9.2) | 99.7 (99.7;99.8) | 74 | 19 | 296 |
| **0.01** | 41.9 (32.4;52.1) | 4.5 (4.2;4.7) | 3.0 (2.2;4.1) | 99.8 (99.7;99.8) | 54 | 39 | 1244 |
| **0.006** | 54.8 (44.7;64.6) | 11.9 (11.5;12.3) | 1.5 (1.2;2.0) | 99.8 (99.8;99.9) | 42 | 51 | 3311 |
| **0.001** | 94.6 (88.0;97.7) | 87.0 (86.6;87.4) | 0.4 (0.3;0.4) | 99.9 (99.7;99.9) | 5 | 88 | 24240 |
| **Model: LDL-C concentration + statin use** | | | | | | | |
| **0.05** | 1.1 (0.2;5.8) | 0.1 (0.1;0.2) | 3.2 (0.6;16.2) | 99.7 (99.6;99.7) | 92 | 1 | 30 |
| **0.02** | 12.9 (7.5;21.2) | 1.1 (1.0;1.2) | 3.8 (2.2;6.5) | 99.7 (99.6;99.8) | 81 | 12 | 304 |
| **0.01** | 38.7 (29.4;48.9) | 5.6 (5.4;5.9) | 2.2 (1.6;3.1) | 99.8 (99.7;99.8) | 57 | 36 | 1574 |
| **0.006** | 52.7 (42.6;62.5) | 14.6 (14.2;15.0) | 1.2 (0.9;1.6) | 99.8 (99.8;99.9) | 44 | 49 | 4067 |
| **0.001** | 90.3 (82.6;94.8) | 84.0 (83.5;84.4) | 0.4 (0.3;0.4) | 99.8 (99.6;99.9) | 9 | 84 | 23393 |

**Supplemental Figure 1. Workflow of the generation of the LDL-C PGS, FH case ascertainment and testing versus training data split of the UK Biobank’s White British participants.** The data was split according to the availability of whole-exome sequencing data. FH = familial hypercholesterolaemia; LDL-C = low-density lipoprotein cholesterol; PGS = polygenic score; QC = quality control; VUS = variants of uncertain significance.


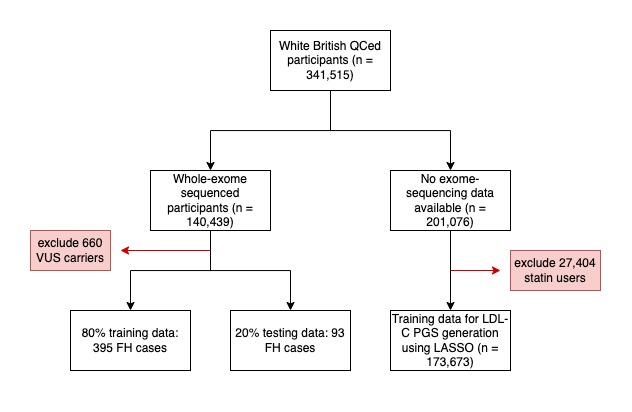


**Supplemental Figure 2. Correlation plot of the variables tested in the LASSO regression model for the prediction of monogenic FH.** The data shown here is from the training dataset as this was used to evaluated highly correlated variables prior to running the LASSO regression. ALP = alkaline phosphatase; ALT = alanine transaminase; Apo-A1 = apolipoprotein A1; ApoB = apolipoprotein B; AST = aspartate aminotransferase; BMI = body mass index; CHD = coronary heart disease; CRP = C-reactive protein; DBP = diastolic blood pressure; FH = familial hypercholesterolaemia; HDL-C = high-density lipoprotein cholesterol; Lp(a) = lipoprotein A; LDL-C = low-density lipoprotein cholesterol; PGS = polygenic score; SBP = systolic blood pressure; T2D = type 2 diabetes.

**
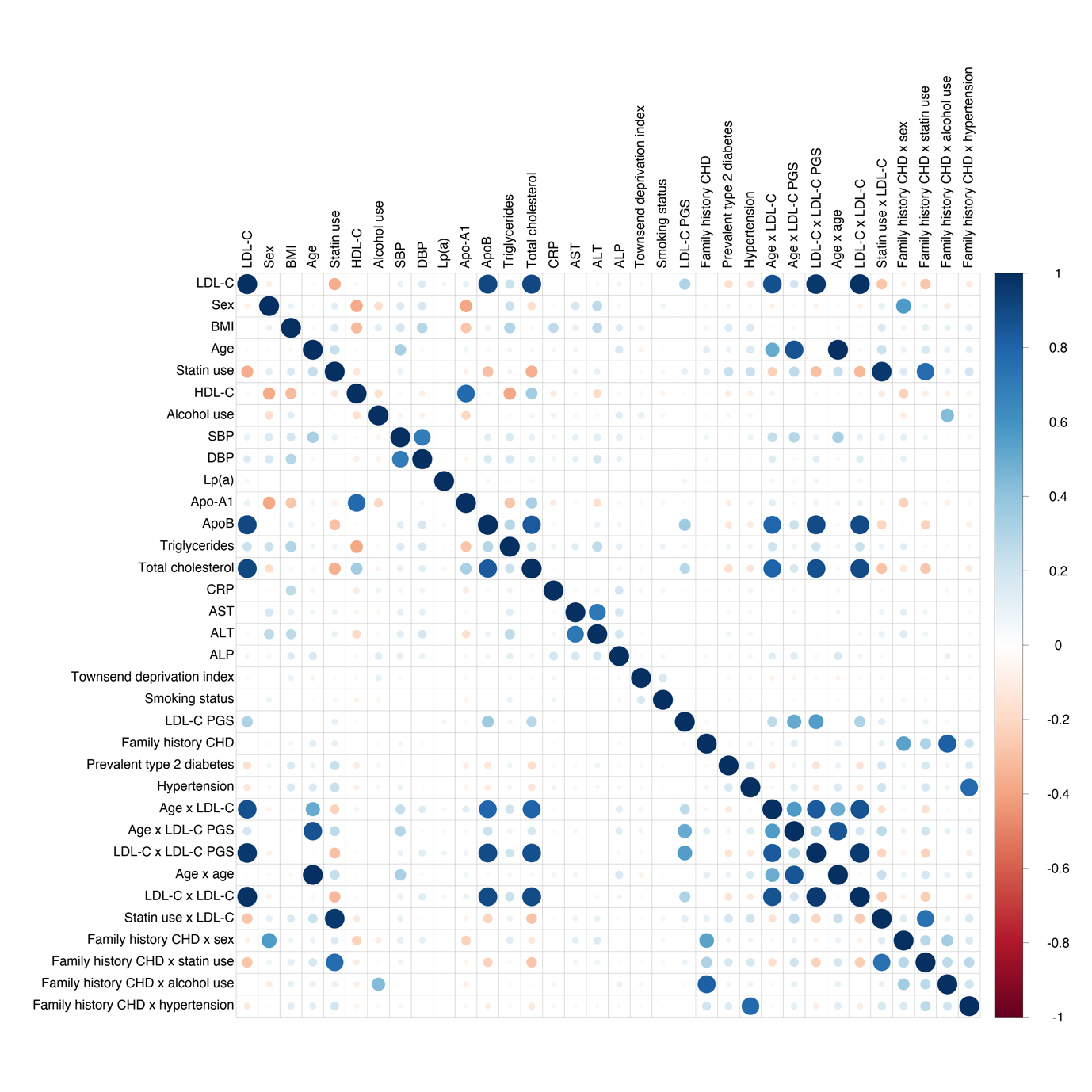
**

**Supplemental Figure 3. LASSO regression model feature selection and importance for monogenic FH prediction.** Feature importance is ordered by value of log odds ratio (OR) per standard deviation (SD). ALT = alanine transaminase; Apo-A1 = apolipoprotein A; BMI = body mass index; CHD = coronary heart disease; CRP = C-reactive protein; DBP = diastolic blood pressure; LDL-C = low-density lipoprotein cholesterol; PGS = polygenic score; T2D = type 2 diabetes.

**
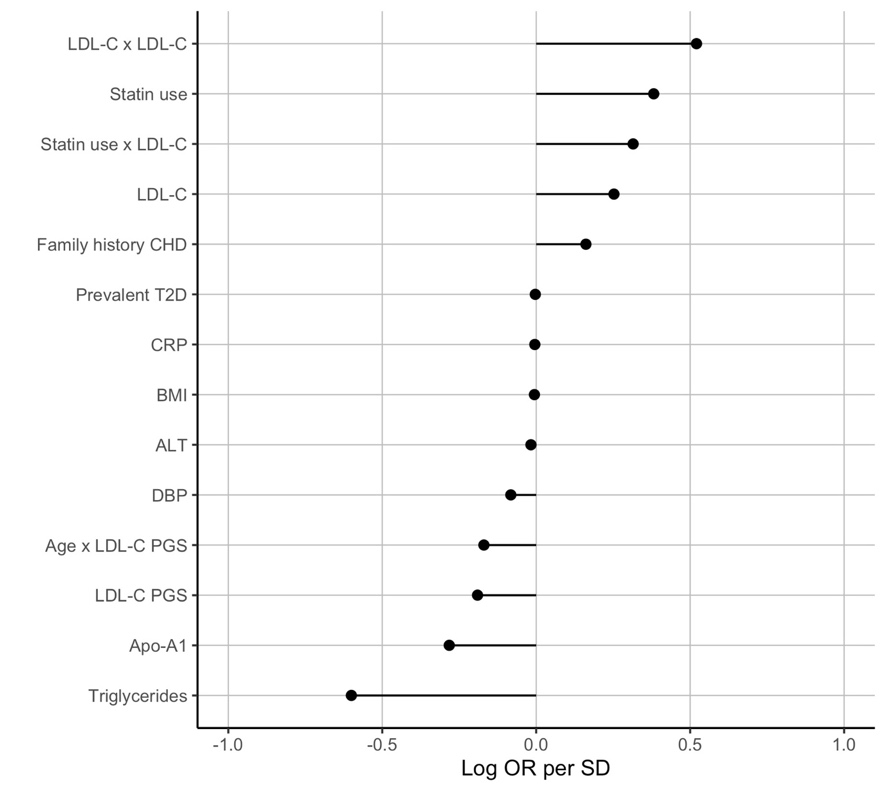
**

**Supplemental Figure 4. The non-linear associations of variables for the prediction of monogenic FH retained by the LASSO model.** LDL-C = low-density lipoprotein cholesterol; FH = familial hypercholesterolaemia.

**
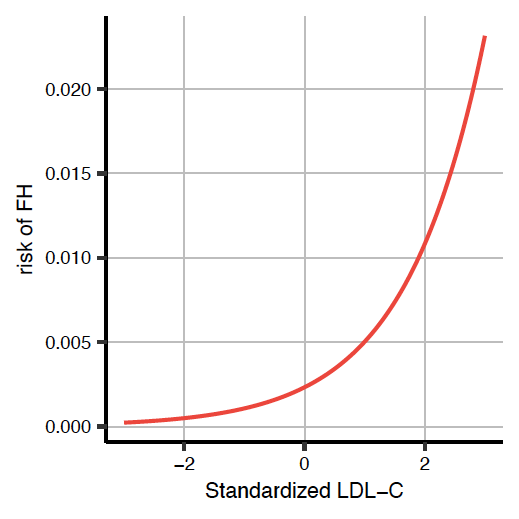
**

**Supplemental Figure 5. The two-stage population screening strategy for monogenic FH.** Stage 1 screen identifies individuals with a predicted probability by the LASSO model above a pre-specified threshold value (in this example: 0.006), followed by a second stage of exome-sequencing. FH cases detected following this two-stage screen are brought forward for cascade testing of first-degree relatives. The sensitivity and false positive rate of the first stage depends on the threshold value chosen for the model. We assume perfect discrimination in the second stage of exome-sequencing (sensitivity of 100% and false positive rate of 0%). Cascade testing is expected to yield 1.5 additional FH cases detected for every FH case identified through the two-stage population screening strategy, as described in the results section. FH = familial hypercholesterolaemia; LDL-C = low-density lipoprotein cholesterol.


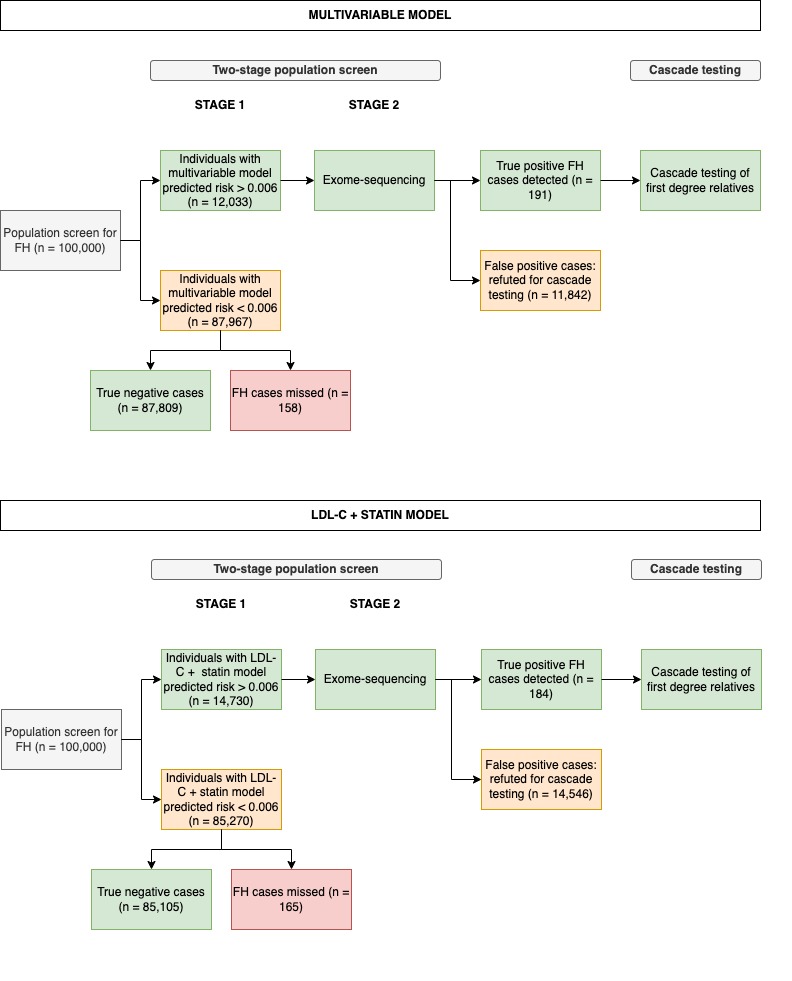

Supplement: Supplementary information [file mmc1.docx]
